# Phylogenomics of “*Candidatus* Hepatoplasma crinochetorum,” a Lineage of Mollicutes Associated with Noninsect Arthropods

**DOI:** 10.1093/gbe/evu020

**Published:** 2014-01-29

**Authors:** Sébastien Leclercq, Jessica Dittmer, Didier Bouchon, Richard Cordaux

**Affiliations:** Université de Poitiers, UMR CNRS 7267 Ecologie et Biologie des Interactions, Equipe Ecologie Evolution Symbiose, Poitiers, France

**Keywords:** *Hepatoplasma*, Mollicutes, genome sequence, symbiont

## Abstract

Bacterial gut communities of arthropods are highly diverse and tightly related to host feeding habits. However, our understanding of the origin and role of the symbionts is often hindered by the lack of genetic information. “*Candidatus* Hepatoplasma crinochetorum” is a Mollicutes symbiont found in the midgut glands of terrestrial isopods. The only available nucleotide sequence for this symbiont is a partial 16S rRNA gene sequence. Here, we present the 657,101 bp assembled genome of *Candidatus* Hepatoplasma crinochetorum isolated from the terrestrial isopod *Armadillidium vulgare.* While previous 16S rRNA gene-based analyses have provided inconclusive results regarding the phylogenetic position of *Candidatus* Hepatoplasma crinochetorum within Mollicutes, we performed a phylogenomic analysis of 127 Mollicutes orthologous genes which confidently branches the species as a sister group to the Hominis group of Mycoplasma. Several genome properties of *Candidatus* Hepatoplasma crinochetorum are also highlighted compared with other Mollicutes genomes, including adjacent tryptophan tRNA genes, which further our understanding of the evolutionary dynamics of these genes in Mollicutes, and the presence of a probably inactivated CRISPR/Cas system, which constitutes a testimony of past interactions between *Candidatus* Hepatoplasma crinochetorum and mobile genetic elements, despite their current lack in this streamlined genome. Overall, the availability of the complete genome sequence of *Candidatus* Hepatoplasma crinochetorum paves the way for further investigation of its ecology and evolution.

Arthropod-associated symbiont communities have been known for a long time, and they show a wide range of interactions from parasitism to mutualism. Bacterial communities associated with arthropod guts are extremely diverse and they generally harbor commensal and nutritional symbionts (Dillon and Dillon 2004). Moreover, the set of symbionts in the community is tightly related to the feeding habits of the host, as some of them are necessary for degradation of host food (Colman et al. 2012). Terrestrial isopods are crustaceans that represent a major component of the litter ecosystem, as they mainly feed on dead plant material and participate in litter decomposition. It is therefore not surprising that terrestrial isopods host a cohort of gut-associated bacteria (Drobne et al. 2002; Kostanjsek et al. 2002, 2007; Wang et al. 2007), in addition to intracellular bacteria such as *Wolbachia* (Bouchon et al. 2008; Cordaux et al. 2011) and *Rickettsiella* (Cordaux et al. 2007).

Initially described in the hepatopancreas (midgut glands) of the terrestrial isopod *Porcellio scaber* (Wang et al. 2004), the extracellular symbiont *Candidatus* Hepatoplasma crinochetorum (hereafter *Hepatoplasma*) is generally found at high frequency in a variety of isopod species, including *P. scaber*, *Oniscus asellus*, *Philoscia muscorum*, *Trachelipus rathkii*, *Ligia oceanica*, *Tylos europaeus*, *Trichoniscus pusillus*, *Alloniscus perconvexus*, and *Armadillidium vulgar*e (Wang et al. 2007; Fraune and Zimmer 2008). *Hepatoplasma* is thought to improve host survival under low nutrient conditions (Fraune and Zimmer 2008) and it may be inherited from parents to offspring through environmental transmission (Wang et al. 2007). However, as yet little is known about the ecology and evolution of this bacterium, and molecular investigations are hindered by the lack of genetic information. This is well illustrated by the fact that phylogenetic relationships of *Hepatoplasma* have been investigated exclusively using the 16S rRNA gene. All phylogenetic analyses concur in placing *Hepatoplasma* within Mollicutes (Wang et al. 2004; Kostanjsek et al. 2007; Fraune and Zimmer 2008; Nechitaylo et al. 2009). The membership of *Hepatoplasma* to Mollicutes is also supported by the total lack of a cell wall (Wang et al. 2004), which is a physiological specificity of Mollicutes (Razin 2006). However, there are still uncertainties on the evolutionary relationships of *Hepatoplasma* relative to the four currently defined groups of Mollicutes: the AAA group (*Acholeplasma*/*Asteroplasma*/*Anaeroplasma*), which also includes plant parasites *Candidatus* Phytoplasma spp.; the Entomoplasmatales group containing insect-associated *Spiroplasma*/*Mesoplasma* species and mammal-associated *Mycoplasma mycoides* subgroup species; and two major groups of *Mycoplasma*, *Hominis* and *Pneumoniae*, which infect various vertebrates including humans (Tully et al. 1993; Razin 2006; Oshima and Nishida 2007). Depending on studies, *Hepatoplasma* has been inferred to be sister to the Pneumoniae group (Wang et al. 2004; Kostanjsek et al. 2007), to the Entomoplasmatales group (Fraune and Zimmer 2008) or to a monophyletic group composed of the Pneumoniae and Hominis groups (Nechitaylo et al. 2009), and generally with little statistical support. To elucidate the phylogenetic relationships of *Hepatoplasma* and provide new molecular tools for the study of the evolution and ecology of these symbionts, we determined the genome sequence of *Hepatoplasma* symbionts from the terrestrial isopod *Armadillidium vulgare* and performed a phylogenomic analysis providing conclusive evidence for *Hepatoplasma* constituting a sister lineage to the Hominis group of Mollicutes.

## Phylogenomic Analyses 

The genome sequence of *Hepatoplasma* strain Av consists of a circular DNA molecule of 657,101 bp with an average G + C content of 22.5% and it harbors a total of 582 CDS, 433 of which have a predicted function (table 1). The genome is highly reduced with a coding density of 94% and only four identified pseudogenes, in line with most other Mollicutes genomes. We conducted a phylogenomic analysis using a set of 127 orthologous gene families concurrently detected in *Hepatoplasma*, 45 available representative Mollicutes genomes, and the three outgroups *Bacillus subtilis*, *Streptococcus pneumoniae*, and *Clostridium perfringens* (see Materials and Methods). Two maximum-likelihood analyses were performed, using the standard LG-I amino acid substitution model (Le and Gascuel 2008) and the Mollicutes-optimized MOLLI60 substitution model (Lemaitre et al. 2011), respectively.

**Table 1:** General Properties of the “*Candidatus* Hepatoplasma crinochetorum” strain Av Genome

Total length (bp)	657,101
GC content (%)	22.5
CDS number	582
Coding density (%)	94
Pseudogenes	4
tRNA number	27
rRNA number	3

Both trees yielded essentially the same topology with few minor branching inconsistencies (supplementary fig. S1, Supplementary Material online). The main inconsistency occurred in the Hominis group, in which the MOLLI60 analysis branched *M. pulmonis* strain with the *M. mobile* strain, while the LG-I analysis branched it at the root of an internal group (including the *M. synoviae* strain, among others). Both topologies were statistically very robust, with bootstrap procedures values slightly higher for the MOLLI60 topology in general (all but one nodes with 100% confidence) than for the LG-I topology (most nodes with 90–100% confidence), suggesting that the former better reflects the true species relationships. Interestingly, the conflicting branching of *M. pulmonis* was previously highlighted in a whole-genome-based Mollicutes phylogeny, in which *M. pulmonis* branched with *M. synoviae* instead of *M. mobile*, with only 61% bootstrap support (Oshima and Nishida 2007). These results emphasize the relevance of using optimized amino acid substitution models to conduct protein sequence comparisons, especially for highly compositionally biased bacterial genomes such as Mollicutes.

**Fig. 1.—:**
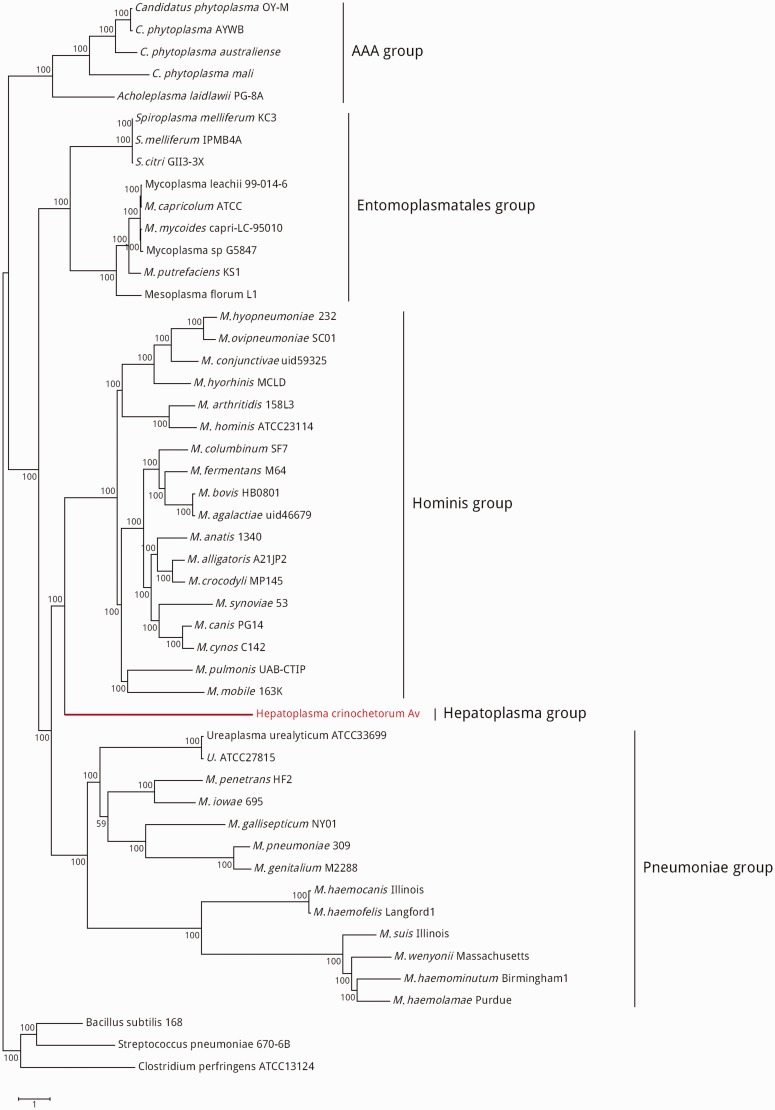
Phylogeny of the 46 Mollicutes strains from which proteomes are available. The tree is based on a set of 127 orthologous proteins and results from a maximum likelihood analysis computed by RAxML using a gamma model of rate heterogeneity, a proportion of invariable sites, and the Mollicutes-optimized substitution matrix MOLLI60. Bootstrap supports were computed using 1,000 iterations and the three Firmicutes *Bacillus subtilis*, *Streptococcus pneumoniae,* and *Clostridium perfringens* were used as outgroups.

Although previous phylogenetic analyses of *Hepatoplasma* exclusively based on 16S rRNA have produced conflicting results (Wang et al. 2004; Kostanjsek et al. 2007; Fraune and Zimmer 2008; Nechitaylo et al. 2009), our phylogenomic approach provides unprecedented support for the branching of *Hepatoplasma* as sister lineage of the Hominis group with 100% confidence under both the LG-I and MOLLI60 models (fig. 1; supplementary fig. S1, Supplementary Material online). From an evolutionary perspective, the nesting of *Hepatoplasma* within a clade grouping the Hominis and Pneumoniae groups (all known strains of which are vertebrate-associated parasites), could at first sight suggest that the ancestor of *Hepatoplasma* was associated to vertebrates and that the presence of this bacterium in arthropods results from a vertebrate-to-invertebrate transfer. However, horizontal transfers of Mollicutes from nonvertebrate to vertebrate hosts have seemingly been common place during Mollicutes evolution. For example, the mammal-infecting subgroup *M. mycoides* originated from insect-associated species (*Spiroplasma* and *Mesoplasma* species) and various *Acholeplasma* and all *Anaeroplasma* strains infecting mammals are phylogenetically related to plant-associated *Phytoplasma* species (Razin 2006). This observation suggests that the alternative hypothesis of an invertebrate-associated *Hepatoplasma* ancestor transferred multiple times to vertebrate hosts remains highly probable. It will be interesting to address this question more comprehensively in the future by adding more invertebrate-associated Mollicutes genomes to the phylogeny.

### Evolution of Tryptophan tRNAs

**Fig. 2.—:**
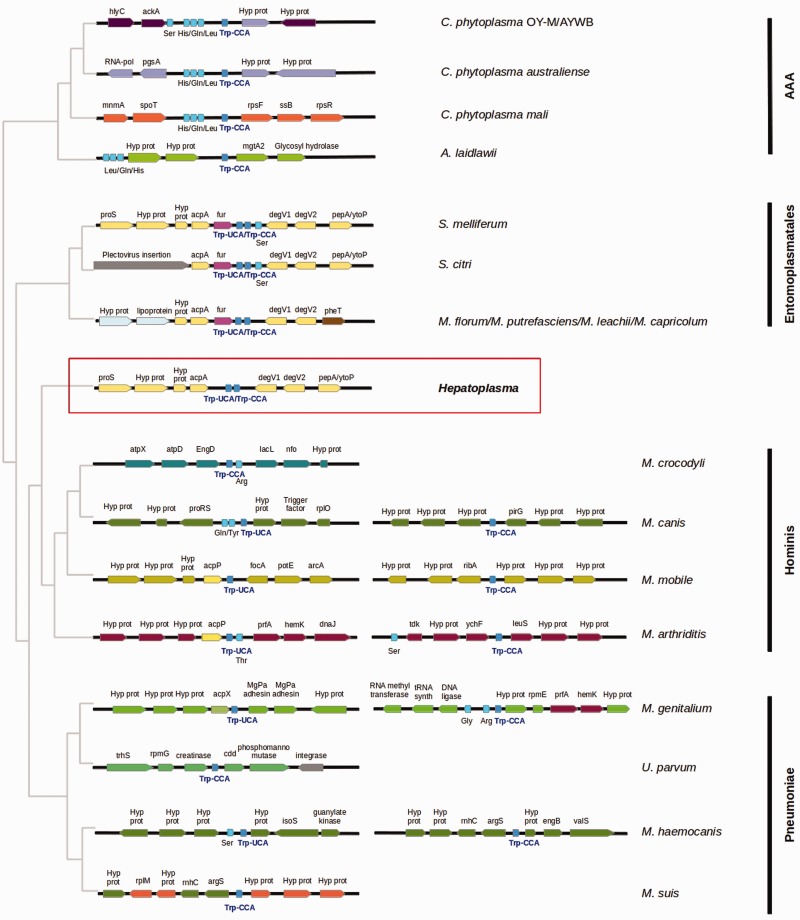
Gene synteny of regions harboring tRNA–Trp in *Hepatoplasma* and different representative Mollicutes genomes. The tRNA anticodon (CCA or UCA) is indicated, and surrounding regions for each tRNA–Trp gene are displayed when the copies are not in tandem. Gene lengths are not to scale. Genes with the same color between genomes are homologous, except for tRNAs which are all colored in light blue, and mobile genetic element genes colored in gray. Phylogenetic relationships displayed on the left are retrieved from figure 1. Phylogenetic group names are indicated on the right. “Hyp prot”: *hypothetical protein*.

The *Hepatoplasma* genome contains a single 5S-23S-16S operon, as well as 27 tRNAs (table 1). Interestingly, both Tryptophan tRNAs (UCA and CCA anticodons) are adjacent, a feature previously detected in *M. capricolum* and *S. citri* (Yamao et al. 1988; Citti et al. 1992). Investigation of tRNA-Trp gene positions in Mollicutes genomes reveals that this feature is restricted to the Entomoplasmatales group in addition to *Hepatoplasma* (fig. 2). Moreover, gene synteny is conserved between the two groups over ∼7 kb, while there is no or very few conservation with the 12 other representative genomes analyzed (fig. 2). According to the aforementioned Mollicutes phylogeny, the occurrence of adjacent tRNA–Trp in both *Hepatoplasma* and Entomoplasmatales suggests that this region has been horizontally transferred between the two groups, or that this gene order is ancestral to all groups but AAA, and subsequent chromosomal rearrangements occurred independently around tRNA–Trp genes in Hominis and Pneumoniae groups.

To investigate these hypotheses, we performed a phylogenetic analysis of the two *degV* genes, which are located immediately downstream of tRNA–Trp genes in Entomoplasmatales and *Hepatoplasma* genomes. Orthologs of these two genes were retrieved in other Mollicutes genomes using the orthogroup information provided by the OrthoMCL procedure (see Materials and Methods), and Neighbor-Joining and ML trees were computed. Both analyses produced congruent trees (supplementary fig. S2, Supplementary Material online), with a well-supported clade for each *degV* gene of Entomoplasmatales and a separate clade for the two *Hepatoplasma degV* genes. Although phylogenetic relationships between these clades are poorly resolved, the monophyly of each *Hepatoplasma* and Entomoplasmatales *degV* clades does not support the hypothesis that *Hepatoplasma degV* genes originate from those of Entomoplasmatales (or vice versa), i.e., shared gene synteny between these two groups probably does not result from horizontal gene transfer.

Yamao et al. (1988) proposed a hypothesis in which a tRNA–Trp with UGA anticodon may appear in bacterial lineages when all (or almost all) TGA termination codons have shifted to TAA, because of the evolution toward AT-biased genomes observed in highly reduced genomes (Moran et al. 2008). In such a case, the tRNA_CCA_ may be duplicated and mutated to tRNA_UCA_ through a C to T mutation in the anticodon, allowing TGG Trp codons present in the genome to mutate to TGA and being still correctly translated. Under this hypothesis, the *Hepatoplasma* tRNA–Trp tandem duplication may represent the ancestral state from which the new anticodon recognition has appeared.

### CRISPR/Cas System

**Fig. 3.—:**
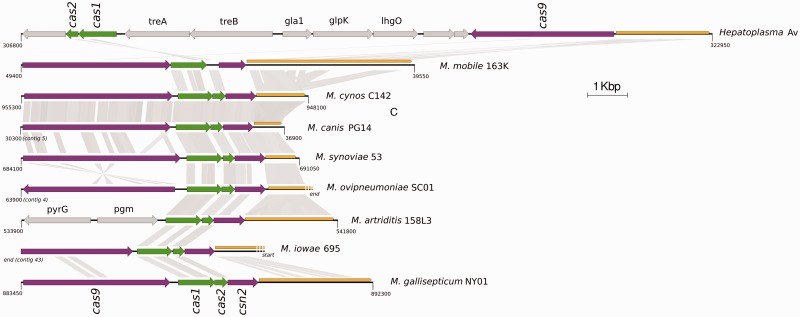
Gene synteny of CRISPR/Cas systems detected in *Hepatoplasma* and eight other Mollicutes genomes. *cas1* and cas2 genes are colored in green, other *cas* genes and the CRISPR locus are colored in purple and orange, respectively. Genes unrelated to the CRISPR/Cas system are colored in gray and their names are indicated when known. TblastX comparisons were performed with an e-value of 10^−9^, and homologous regions larger than 40 bp are indicated with gray frames. CRISPR loci of *Mycoplasma iowae* and *M. ovipneumoniae* are artificially truncated because of the contig ends.

The *Hepatoplasma* genome contains no detectable phage, plasmid, transposon, insertion sequence, or group I or II intron gene. The absence of mobile genetic elements is a common feature of streamlined bacterial symbiont genomes (Moran et al. 2008; Moya et al. 2008), although notorious exceptions exist such as *Wolbachia* (Cerveau et al. 2011; Leclercq et al. 2011). Interestingly, we detected a CRISPR/Cas system using the CRISPRFinder interface (Grissa et al. 2007). CRISPR/Cas systems are molecular systems involved in defense of prokaryotes against phage and other mobile genetic element infections (Deveau et al. 2010). CRISPR/Cas systems are composed of a CRISPR locus, and a variable number of CRISPR-associated (*cas*) genes located in the vicinity of the CRISPR locus (Deveau et al. 2010; Horvath and Barrangou 2010). Several CRISPR/Cas subtypes have been described, each with a specific subset of *cas* genes (Makarova et al. 2011). In *Hepatoplasma*, the CRISPR locus is located at genome coordinates 320,594–322,939 and consists of 35 spacers separated by 36-bp repeat units. The genome harbors *cas1* and *cas2* genes, considered as universal markers of CRISPR/Cas systems, and the *cas9* gene, which is typical of the type II CRISPR/Cas subtype (Makarova et al. 2011). The type II CRISPR/Cas subtype is usually composed of *cas9* (formerly *csn1*), *cas1*, *cas2*, and *csn2* (for type IIA) or *cas4* (for type IIB) genes, all located in a single transcriptional unit directly upstream of the CRISPR locus. However, in *Hepatoplasma*, neither *csn2* nor *cas4* is present, *cas9* is in reverse orientation compared with the CRISPR locus, and c*as1* and c*as2* are located 13 kb upstream of the CRISPR locus, in reverse orientation, and separated from *cas9* by 7 genes involved in other metabolic functions (fig. 3). Contrary to *Hepatoplasma*, the eight other Mollicutes genomes in which we found CRISPR/Cas systems all show the typical type IIA subtype organization (with some minor rearrangements/pseudogenization in *M. mobile*, *M. ovipneumoniae*, and *M. arthriditis*), indicating that the unusual organization we recorded in *Hepatoplasma* does not reflect a general feature of CRISPR/Cas systems in Mollicutes (fig. 3; supplementary table S1, Supplementary Material online). Previous studies on the *Streptococcus thermophilus* type IIA CRISPR/Cas subtype suggest that *csn2* is involved in the adaptation stage of immunity acquisition, that is, the acquisition of invaders’ DNA fragments used to prevent further infections (Barrangou et al. 2007; Garneau et al. 2010). Bacterial strains devoid of this gene, such as *Hepatoplasma*, may thus not update their defense against new infections with mobile genetic elements, which strongly reduces the efficiency of the CRISPR/Cas system. Therefore, the CRISPR/Cas system in *Hepatoplasma* may have become largely inefficient, allowing c*as* gene rearrangements without counterselection. Altogether, these observations suggest that the CRISPR/Cas system has likely been inactivated in *Hepatoplasma*.

It is noteworthy that despite the absence of *csn2*, the *cas9*, *cas1*, and *cas2* genes do not show any apparent hallmark of pseudogenization and all 35 repeat units of the CRISPR locus are strictly identical, suggesting that the CRISPR/Cas system, if inactive, was inactivated recently. In any event, the occurrence of a CRISPR-Cas system in the *Hepatoplasma* genome, even though it is probably nonfunctional, reveals that *Hepatoplasma* has been confronted to mobile genetic elements in its evolutionary past. Therefore, the current lack of mobile genetic elements in this genome does not reflect that it has never had any. Rather, it indicates that remnants of past invasions have been eliminated through the process of reductive evolution that symbiont genomes usually experience (Moran et al. 2008; Moya et al. 2008).

## Conclusion

Whole-genome-based phylogenies have proven their usefulness to disentangle ambiguous or inconclusive phylogenetic relationships provided by single gene analyses (Zhao et al. 2005; Oshima and Nishida 2007; Naushad and Gupta 2013). In this study, we obtained the complete genome sequence of *Hepatoplasma* and used it to resolve its phylogenetic position within Mollicutes. The availability of a full genome sequence also allowed us to further our understanding of the tryptophan tRNA genes evolutionary dynamics in Mollicutes. Finally, the presence of a probably inactivated CRISPR system constitutes a testimony of past interactions between *Hepatoplasma* and mobile genetic elements, despite their current lack in this streamlined genome. This novel genomic resource will fuel studies aiming to better understand the ecological and evolutionary properties of this invertebrate-associated group of Mollicutes.

## Materials and Methods

### Genome Sequencing and Assembly

The *Hepatoplasma* genome from the terrestrial isopod *A. vulgare* was assembled from data generated as part of the ongoing *A. vulgare* genome project (Leclercq S and Cordaux R, unpublished data). Briefly, total genomic DNA was extracted from a single *A. vulgare* individual. A paired-end library with ∼370 bp inserts was prepared and sequenced on an Illumina HiSeq2000. Reads were filtered with FastQC and assembled using the SOAP de novo software version 1.05. The best assembly (obtained with a *k*-mer size of 49) was screened for *Hepatoplasma*-derived sequences using the proteomes of *Mesoplasma florum* L1, *M. agalactiae* ASM8986v1, *M. suis* Illinois, *Spiroplasma melliferum* IPMB4A, *S. melliferum* KC3, and *Ureaplasma urealyticum* ATCC27618 downloaded from the NCBI FTP website (ftp://ftp.ncbi.nlm.nih.gov/genomes/Bacteria/, last accessed February 14, 2014) as queries for a BlastX search (minimal identity of 37% over 80% of the query protein size), to identify contigs containing Mollicutes-like genes. Twenty-four contigs stood out of the analysis as likely *Hepatoplasma* contigs because of an atypical but consistently homogeneous coverage of 350× or more (40× on average for *A. vulgare* contigs). These 24 contigs were subsequently concatenated into 3 contigs with SSPACE version 2.0 (Boetzer et al. 2011), which were then assembled into a circular sequence of 657,101 bp using reads from a mate-pair library with ∼4,100 bp inserts sequenced on an Illumina HiSeq2000 and several GapFiller iterations (Boetzer and Pirovano 2012). Assembly errors were experimentally corrected and validated using PCR and Sanger sequencing at the junctions between the 24 initial contigs, and through mapping of the whole paired-end reads set onto the reconstructed sequence. The genome sequence of *Hepatoplasma* strain Av is available under the GenBank accession number CP006932.

### Genome Annotation

Annotation was performed using the Prokka annotation pipeline version 1.5.2 (Prokka: Prokaryotic Genome Annotation System—http://vicbioinformatics.com/, last accessed February 14, 2014). Parameters “genus” and “genetic code” were set to “Mycoplasma” and “4,” respectively, to fit with the properties of Mollicutes genomes. Prokka executed Aragorn 1.2.34, RNAmmer 1.2, Prodigal 2.60, and HMMER3/BlastP to retrieve tRNAs, mRNAs, open reading frames, and gene annotations, respectively. Pseudogenes were detected using the ψ–φ program suite (Lerat and Ochman 2004) with all other Mollicutes genomes as references. Because of the specific Mollicutes genetic code (not implemented in ψ–φ) and high genetic distance between *Hepatoplasma* and other Mollicutes, many detected pseudogenes were false positives and were manually curated. Genes related to mobile genetic elements were searched in annotated genes using keywords “transposase/Tpase” and “IS” for insertion sequences; “phage/prophage,” “integrase,” and “recombinase” for prophages, transposons, and group I introns; “reverse-transcriptase” and “RNA-directed DNA polymerase” for group II introns.

### Orthology and Phylogenomic Analyses

A set of 30 representative Mollicutes proteomes was downloaded from the NCBI FTP website (ftp://ftp.ncbi.nlm.nih.gov/genomes/Bacteria/, last accessed February 14, 2014) on March 21, 2013, and 15 additional proteomes were retrieved directly from NCBI Genome Projects. Proteomes of *Bacillus subtilis* 168, *Streptococcus pneumoniae* 670-EB, and *Clostridium perfringens* ATCC 13124 were also downloaded as outgroups.

Orthology relationships among all proteomes (*Hepatoplasma* and the 48 other species) were inferred using the OrthoMCL pipeline (Li et al. 2003), based on all-against-all BlastP hits with an e-value of 10^−^^3^. Among the 5,392 orthology groups returned, 377 contained at least one *Hepatoplasma* protein.

Phylogenomic analyses were performed on a subset of 127 orthogroups harboring at least 40 orthologs including one in *Hepatoplasma* and in each of the three outgroups, and not containing any paralog in any taxon. Proteins of each orthogroup were aligned independently using MAFFT v.7.037 (Katoh et al. 2005) with the Mollicutes-optimized MOLLI60 substitution matrix (Lemaitre et al. 2011), available at (http://www.biomedcentral.com/1471-2105/12/457/additional, last accessed February 14, 2014). Resulting alignments were then concatenated in a single alignment of 46,607 residues, in which missing proteins in some orthogroup alignments were replaced by tracks of gaps.

To select the model of protein evolution that best fitted our Mollicutes data set, we performed a PROTTEST 3 preanalysis (Abascal et al. 2005). The program returned the LG model (Le and Gascuel 2008) with a gamma model of rate heterogeneity, a proportion of invariable sites, and an empirical residue frequency as the best model. RAxML (Stamatakis 2006) was used to compute maximum likelihood trees in two ways. First, we ran the algorithm using the PROTGAMMAILGF amino acid model, which means that the estimation follows a gamma model of rate heterogeneity (four discrete rate categories, and all parameters estimated by RAxML), with the LG substitution matrix, a proportion of invariable sites, and uses empirical residue frequency. The second run was performed under the same gamma model of rate heterogeneity and empirical residue frequency, but using the earlier-mentioned MOLLI60 substitution matrix. Branching confidence was estimated with 1,000 bootstraps for each run.

### Tryptophan tRNA Analysis

Tryptophan tRNA positions were retrieved in each genome by a manual search of GenBank annotation files. tRNA–Trp sequences were extracted and aligned with MAFFT (“linsi” option) to the described tRNA–Trp of *M. capricolum* (Yamao et al. 1988), to assign the correct anticodon sequence to each tRNA. Genes surrounding each tRNA–Trp were investigated through visual inspection under the GenBank graphical interface. *DegV* orthologs were extracted from all proteomes using orthology information returned by the OrthoMCL procedure. The 66 resulting sequences were aligned using the “linsi” option of MAFFT and the MOLLI60 substitution matrix. A maximum likelihood tree was computed using RAxML with the same options as for the global phylogenomic analysis (see above), and a Neighbor-Joining tree was computed using Mega 5.1 (Tamura et al. 2011), with the JTT model of substitution, a gamma model of rate heterogeneity, a pairwise deletion comparison, and 500 bootstrap iterations.

### CRISPR/Cas System Detection

*Cas* genes were first retrieved from Mollicutes genomes using GenBank annotations. *Hepatoplasma*, *M. canis*, *M. cynos*, *M. gallisepticum*, *M. iowae*, and *M. ovipneumoniae* were found to harbor *cas* genes. Orthologous genes in other genomes were then recovered from orthogroups returned by OrthoMCL, which added *M. synoviae*, *M. arthriditis*, and *M. mobile* to the list of Mollicutes harboring *cas* genes. For these nine genomes, a CRISPR region was searched by using the CRISPRFinder webpage (Grissa et al. 2007). A single CRISPR region was detected in every genome, and their position relative to the *cas* genes was then investigated through visual inspection under the GenBank graphical interface. The CRISPR/Cas comparison figure was created with EasyFig version 2.1 (Sullivan et al. 2011).

## Supplementary Material

Supplementary figures S1 and S2 and table S1 are available at *Genome Biology and Evolution* online (http://www.gbe.oxfordjournals.org/).

Supplementary Data
