# Supplementary material for: Phylogenomics of “Candidatus Hepatoplasma crinochetorum,” a Lineage of Mollicutes Associated with Noninsect Arthropods
Source: Genome Biol Evol. 2014 Jan 29;6(2):407–15. doi: 10.1093/gbe/evu020 (PMC3942034; doi:10.1093/gbe/evu020)
Supplement: Supplementary Data [file supp_6_2_407__index.html]

Phylogenomics of 'Candidatus Hepatoplasma crinochetorum', a lineage of Mollicutes associated with non-insect arthropods — Phylogenomics of “Candidatus Hepatoplasma crinochetorum,” a Lineage of Mollicutes Associated with Noninsect Arthropods — Supplementary Data 

# Phylogenomics of “*Candidatus* Hepatoplasma crinochetorum,” a Lineage of Mollicutes Associated with Noninsect Arthropods

## Supplementary Data

files

**Files in this Data Supplement:**

- Supplementary Data - jpeg file
- Supplementary Data - jpeg file
- Supplementary Data - xls file
