# Supplementary figures and images for: Phylogenomics of “Candidatus Hepatoplasma crinochetorum,” a Lineage of Mollicutes Associated with Noninsect Arthropods
Source: Genome Biol Evol. 2014 Jan 29;6(2):407–15. doi: 10.1093/gbe/evu020 (PMC3942034; doi:10.1093/gbe/evu020)

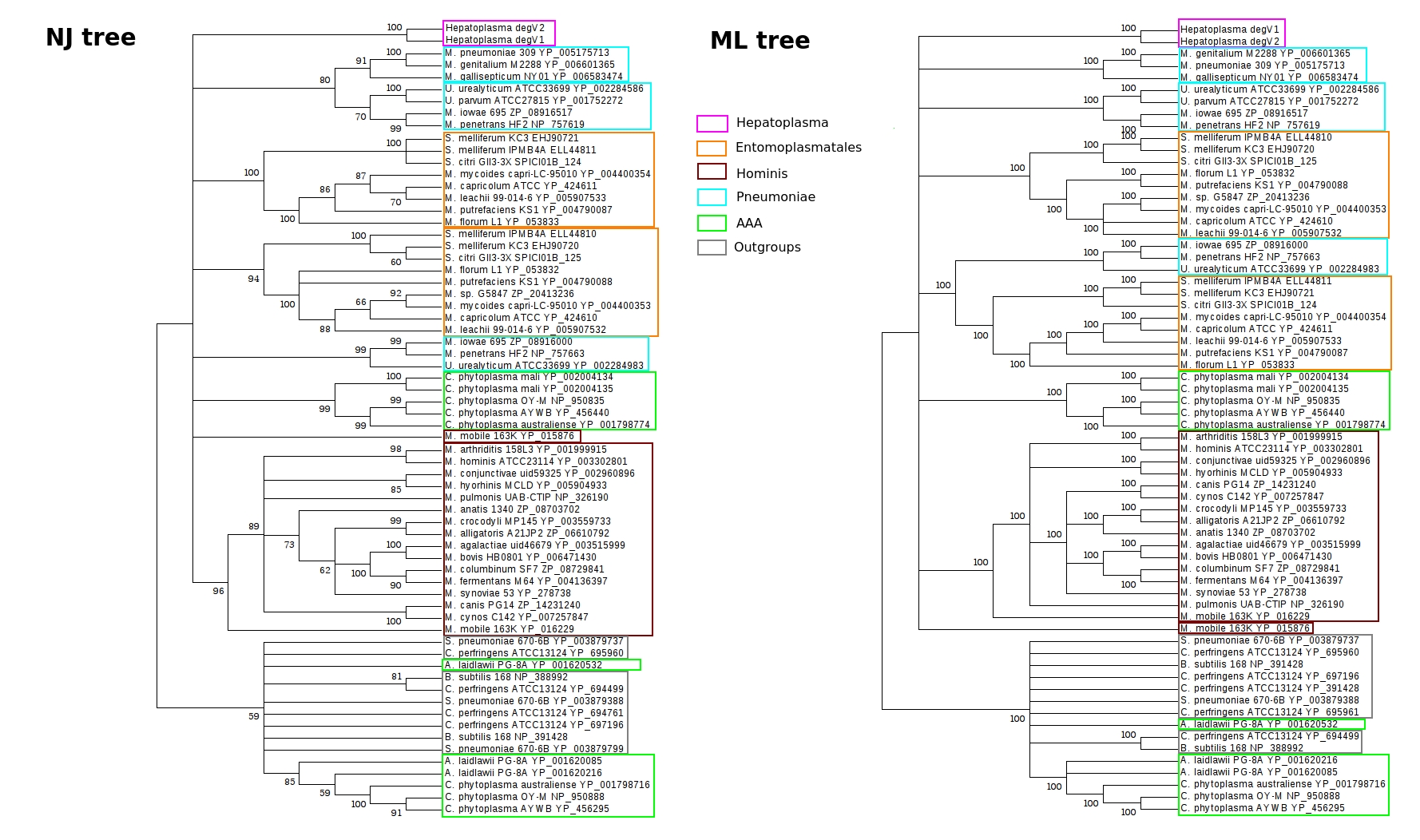

Supplement: Supplementary Data [file supp_evu020_Suppl_Fig2.jpg]

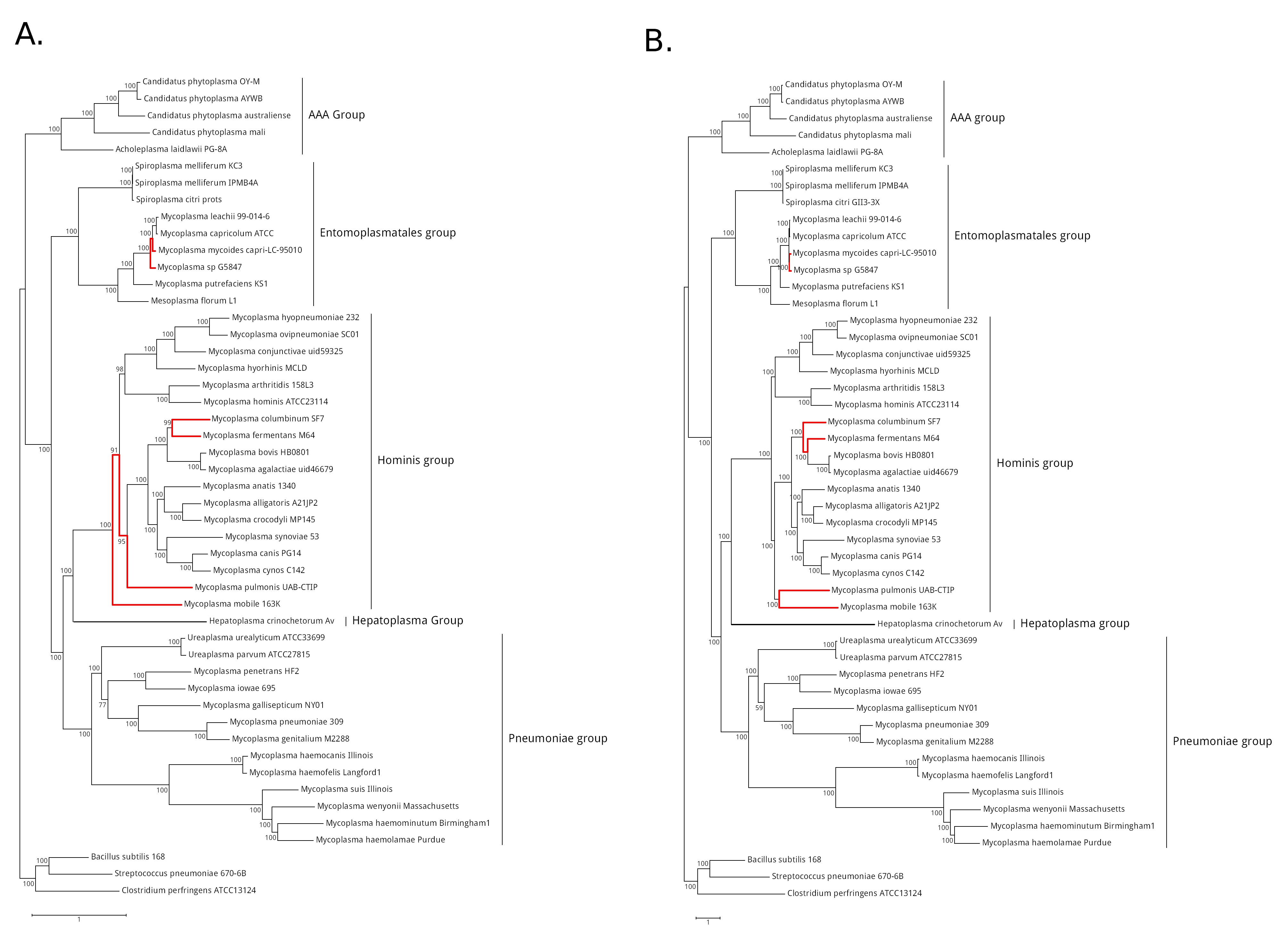

Supplement: Supplementary Data [file supp_evu020_Suppl_Fig_1.jpg]
